# Supplementary material for: Low temperature self-densification of high strength bulk hexagonal boron nitride
Source: Nat Commun. 2019 Feb 20;10:854. doi: 10.1038/s41467-019-08580-9 (PMC6382832; doi:10.1038/s41467-019-08580-9)
Supplement: Supplementary file 1 — Supplementary Information [file 41467_2019_8580_MOESM1_ESM.pdf]

Supplementary Information

Low temperature self-densification of high strength bulk hexagonal boron nitride

Yang *et al.*

Supplementary Note 1: Phase transformation of cBN-hBN

In boron nitride system, there are four primary crystalline phases that include the hexagonal (hBN), rhombohedral (rBN), cubic zinc-blende (cBN), and wurtzite (wBN) forms[1]. Previous experimental and theoretical studies have consistently shown that cBN is the thermodynamically stable phase in ambient conditions[2–4]. The phase transition temperature of cBN-hBN at atmospheric pressure is reported between 1350 and 1800 K[5-7], with a wide temperature range for the full conversion. Phase *p,T* diagrams of boron nitride were calculated by Solozhenko[8-10].

Some of the main recent results on BN are as follows:

1. Petrusha[11] and Sachdev[12] demonstrated the transformation takes place from the cBN particle surface.

2. The onset temperature of the phase transformation from cBN to hBN depends significantly on grain size[12]. Fine-grained samples show a significantly lower conversion temperature than coarse material.

3. Impurities also affect the phase transformation temperature of cBN. Al_2O_3[13] will effectively lower the phase transition temperature of cBN, while the transition temperature will rise with SiO_2[14] and SiAlON[15] added.

Supplementary Note 2: The enthalpy at different temperatures

The enthalpy at different temperatures can be calculated by:

$$\Delta_{\mathrm{tr}}H_{\mathrm{c-h}}(T) = -\Delta_{\mathrm{tr}}H^{0}_{\mathrm{h-c}} + \int_{298.15}^{T} \Delta C_p^{\mathrm{hBN}}(T)\, dT - \int_{298.15}^{T} \Delta C_p^{\mathrm{cBN}}(T)\, dT \qquad (1)$$

where T is the temperature in Kelvin, and $\Delta_{\mathrm{tr}}H^{0}_{\mathrm{h-c}}$= -16.2 kJ mol^{-1} is the enthalpy change of the hBN-cBN phase transition at standard temperature and pressure[9]. $\Delta C_p^{\mathrm{hBN}}(T)$ and $\Delta C_p^{\mathrm{cBN}}(T)$ are the heat capacities of hBN and cBN as functions of temperature, respectively, which can be represented by an adaptive pseudo-Debye model[16,17]:

$$\Delta C_p(T) = 3R\tau^3 \frac{4C_0 + 3C_1\tau + 2C_2\tau^2 + \tau^3}{(C_0 + C_1\tau + C_2\tau^2 + \tau^3)^2}[1 + A\frac{\tau^4}{(0.125 + \tau)^3}] \qquad (2)$$

where R is the gas constant. $\tau = T/\Theta$ denotes the reduced temperature, and Θ corresponds to the high-temperature value of the usual Debye temperature[3]. C_0, C_1, C_2, and A are free parameters given by Solozhenko[10].

Supplementary Note 3: Models of hBN flakes and BN onions

The models of hBN flake and rounded BN onion were built by SolidWorks software (Supplementary Figure 1).

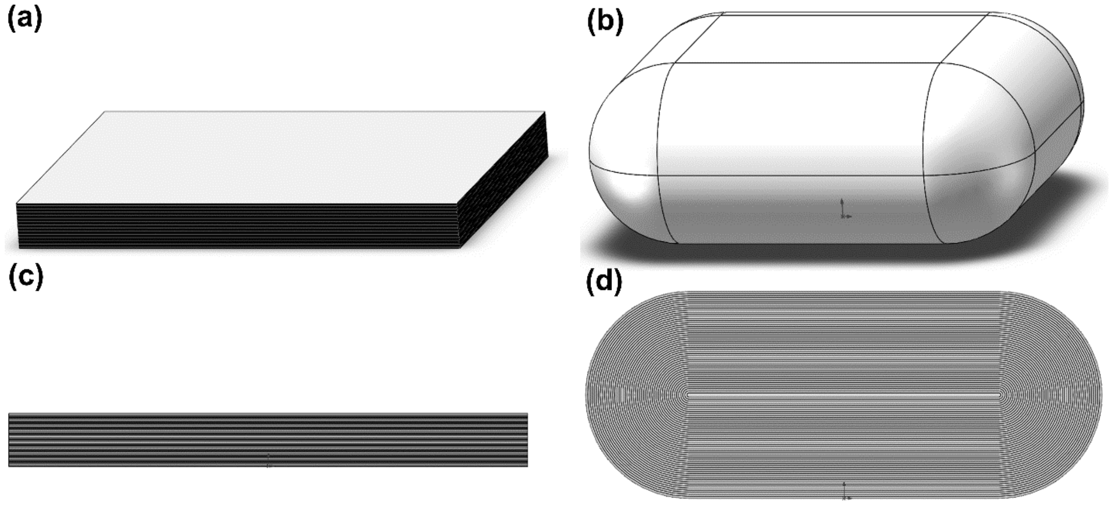

Supplementary Figure 1 Models of hBN flake (a) and BN onion (b); Cross section of hBN flake (c) and BN onion (d).

Due to software size limitations, we scaled the model proportionally. Each layer was 0.33 mm thick as the model of a BN single layer (0.33 nm). Fifty layers, 200×160×16.5 mm cuboid, were built as the model of a single hBN flake (Supplementary Figure 1a, c), and 50 layers, 90×72×36 mm rounded concentric cuboid were built as the model of a single BN onion (Supplementary Figure 1b, d).

Supplementary Note 4: FESEM micrographs on polished surfaces

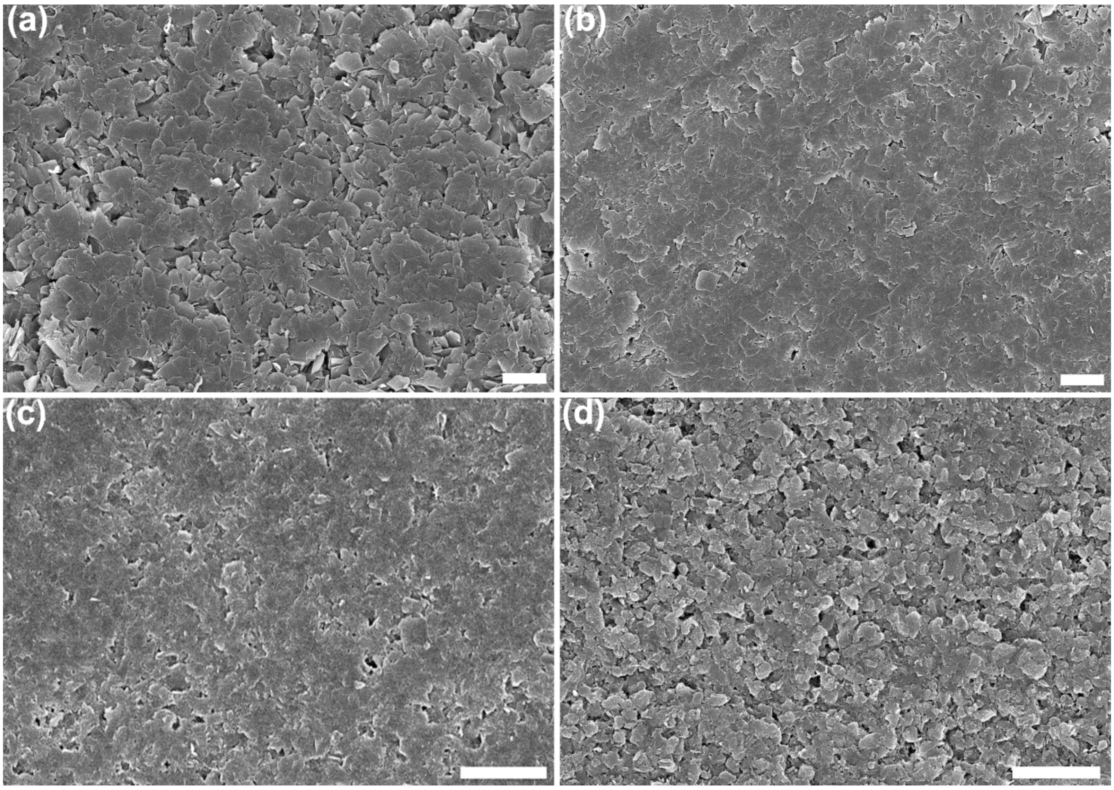

Supplementary Figure 2 FESEM micrographs on polished surfaces of hBN ceramics (a), HSHBN-30 (b), HSHBN-80 (c), HSHBN-100 (d). All scale bars are 1 μm.

The HSHBN-30 has the highest density (Supplementary Figure 2b), and the relative density reaches 97.6%.

Supplementary Note 5: Thermal and dielectric properties

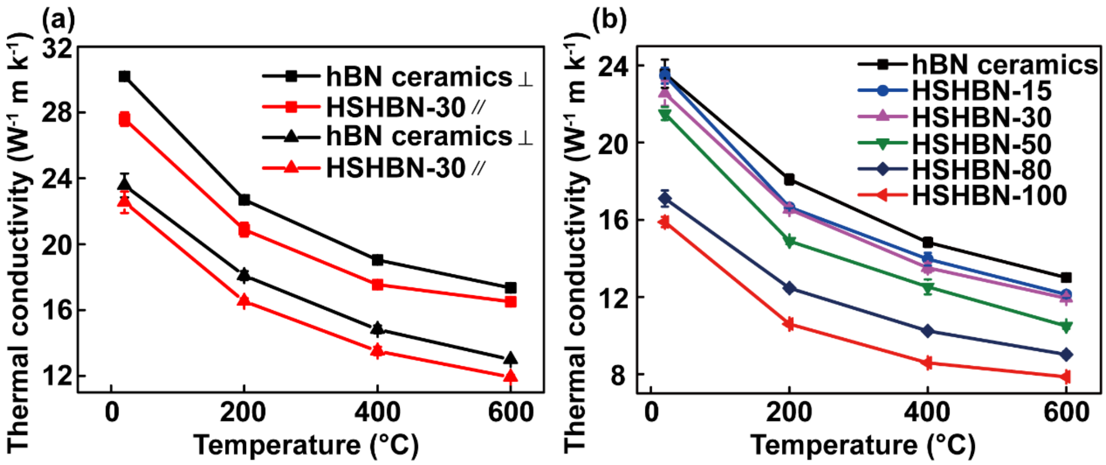

Supplementary Figure 3 Thermal conductivities of pure hBN ceramics and HSHBN-30 sample parallel and perpendicular to the hot-pressing direction (a), and thermal conductivities of samples in the hot-pressing direction (b).

The thermal conductivities of pure hBN ceramics and HSHBN samples were derived from the thermal diffusivity measured by a laser flash thermal analyzer (LFA, Linseis PD1000). To measure the thermal conductivities perpendicular to the hot-pressing direction, the pellets were first cut into bars and rotated 90° to make the section face up, then glued together by applying a thin layer of high-temperature glue, to form a new pellet[18].

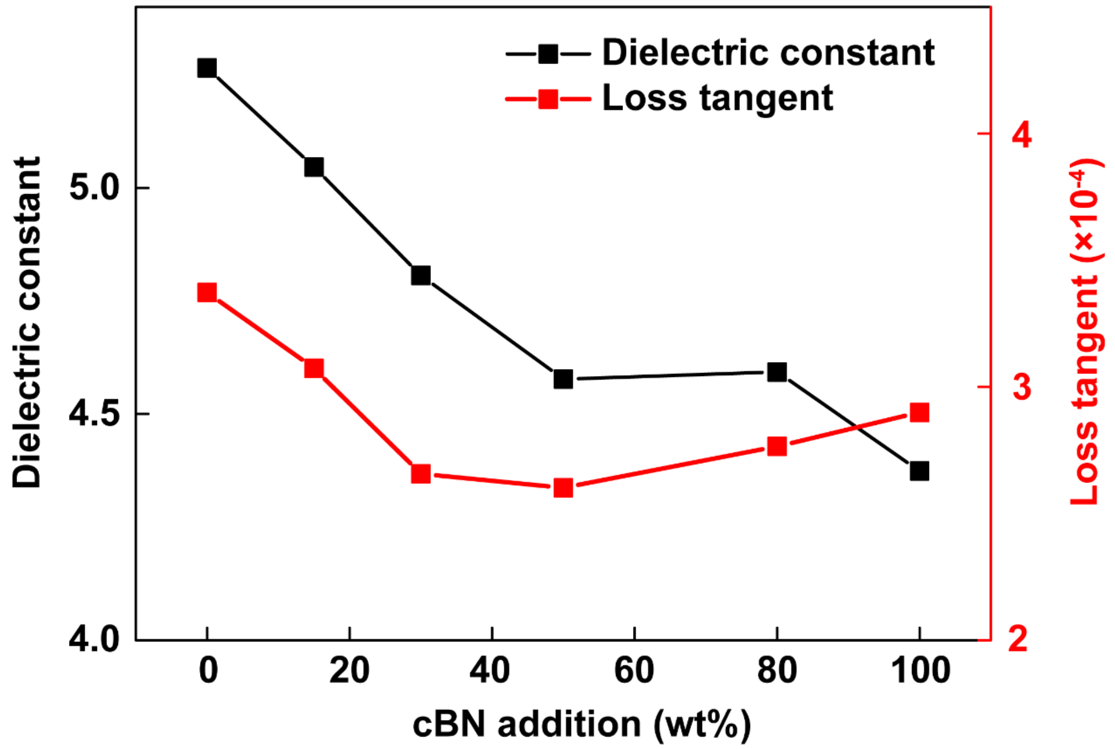

Supplementary Figure 4 Dielectric constant and dielectric loss tangent of HSHBN ceramics with different cBN additions.

The dielectric constant and dielectric loss tangent of samples were measured by a network analyzer (Agilent N5230A,) at the frequency range of 8-18 GHz.

Supplementary References

1. Yu, W., Lau, W., Chan, S., Liu, Z. & Zheng, Q. Ab initio study of phase transformations in boron nitride. *Phys. Rev. B* **67,** 014108 (2003).

2. Bohr, S., Haubner, R. & Lux, B. Comparative aspects of c-BN and diamond CVD. *Diam. Relat. Mater.* **4,** 714–719 (1995).

3. Kern, G., Kresse, G. & Hafner, J. Ab initio calculation of the lattice dynamics and phase diagram of boron nitride. *Phys. Rev. B* **59,** 8551–8559 (1999).

4. Ohba, N., Miwa, K., Nagasako, N. & Fukumoto, A. First-principles study on structural, dielectric, and dynamical properties for three BN polytypes. *Phys. Rev. B* **63,** 115207 (2001).

5. Solozhenko, V. & Turkevich, V. Thermoanalytical study of the polymorphic transformation of cubic into graphite-like boron nitride. *J. Therm. Anal.* **38,** 1181–1188 (1992).

6. Milledge, H., Nave, E. & Weller, F. Transformation of Cubic Boron Nitride to a Graphitic Form of Hexagonal Boron Nitride. *Nature* **184,** 715–715 (1959).

7. Gavrichev, K. *et al.* Low-temperature heat capacity and thermodynamic properties of four boron nitride modifications. *Thermochim. Acta* **217,** 77–89 (1993).

8. Solozhenko, V. New concept of BN phase diagram: an applied aspect. *Diam. Relat. Mater.* **4,** 1–4 (1994).

9. Solozhenko, V. Boron nitride phase diagram. State of the art. *High Press. Res.* **13,** 199–214 (1995).

10. Solozhenko, V., Turkevich, V. & Holzapfel, W. Refined Phase Diagram of Boron

Nitride. *J. Phys. Chem. B* **103,** 2903–2905 (1999).

11. Petrusha, I. Features of a cBN-to-graphite-like BN phase transformation under pressure. *Diam. Relat. Mater.* **9,** 1487–1493 (2000).

12. Sachdev, H., Haubner, R., Nöth, H. & Lux, B. Investigation of the c-BN/h-BN phase transformation at normal pressure. *Diam. Relat. Mater.* **6,** 286–292 (1997).

13. Hotta, M. & Goto T. Densification and microstructure of Al_2O_3-cBN composites prepared by spark plasma sintering. *J. Ceram. Soc. Japan* **116,** 744–748 (2008).

14. Zhang, J., Tu, R. & Goto, T. Densification, microstructure and mechanical properties of SiO2–cBN composites by spark plasma sintering. *Ceram. Int.* **38,** 351–356 (2012).

15. Hotta, M. & Goto, T. Densification and Phase Transformation of β-SiAlON-Cubic Boron Nitride Composites Prepared by Spark Plasma Sintering. *J. Am. Ceram. Soc.* **92,** 1684–1690 (2009).

16. Holzapfel, W. Approximate equations of state for solids from limited data sets. *J. Phys. Chem. Solids* **55,** 711–719 (1994).

17. Holzapfel, W. Equations of state for strong compression. *High Press. Res.* **7,** 290–292 (1991).

18. Mateti, S. *et al.* Bulk Hexagonal Boron Nitride with a Quasi-Isotropic Thermal Conductivity. *Adv. Funct. Mater.* **28,** 1707556 (2018).
